# A Critical Appraisal of COVID-19 in Malaysia and Beyond

**DOI:** 10.21315/mjms2020.27.2.1

**Published:** 2020-04-10

**Authors:** Jafri Malin Abdullah, Wan Faisham Nu’man Wan Ismail, Irfan Mohamad, Asrenee Ab Razak, Azian Harun, Kamarul Imran Musa, Yeong Yeh Lee

**Affiliations:** 1 Editorial Board Member, Malaysian Journal of Medical Sciences, School of Medical Sciences, Universiti Sains Malaysia, Kelantan, Malaysia; 2 Brain Behaviour Cluster and Department of Neurosciences, School of Medical Sciences, Universiti Sains Malaysia, Kelantan, Malaysia; 3 Department of Orthopaedics, School of Medical Sciences, Universiti Sains Malaysia, Kelantan, Malaysia; 4 Department of Otorhinolaryngology-Head & Neck Surgery, School of Medical Sciences, Universiti Sains Malaysia, Kelantan, Malaysia; 5 Department of Psychiatry, School of Medical Sciences, Universiti Sains Malaysia, Kelantan, Malaysia; 6 Department of Medical Microbiology & Parasitology, School of Medical Sciences, Universiti Sains Malaysia, Kelantan, Malaysia; 7 Department of Community Medicine, School of Medical Sciences, Universiti Sains Malaysia, Kelantan, Malaysia; 8 Department of Medicine, School of Medical Sciences, Universiti Sains Malaysia, Kelantan, Malaysia

**Keywords:** COVID-19, SARS-CoV-2, Malaysia, technology, medicine, appraisal

## Abstract

When the first report of COVID-19 appeared in December 2019 from Wuhan, China, the world unknowingly perceived this as another flu-like illness. Many were surprised at the extreme steps that China had subsequently taken to seal Wuhan from the rest of the world. However, by February 2020, the SARS-CoV-2 virus, which causes COVID-19, had spread so quickly across the globe that the World Health Organization officially declared COVID-19 a pandemic. COVID-19 is not the first pandemic the world has seen, so what makes it so unique in Malaysia, is discussed to avoid a future coronacoma.

## The New Year with New Pandemic

**Figure 1:**
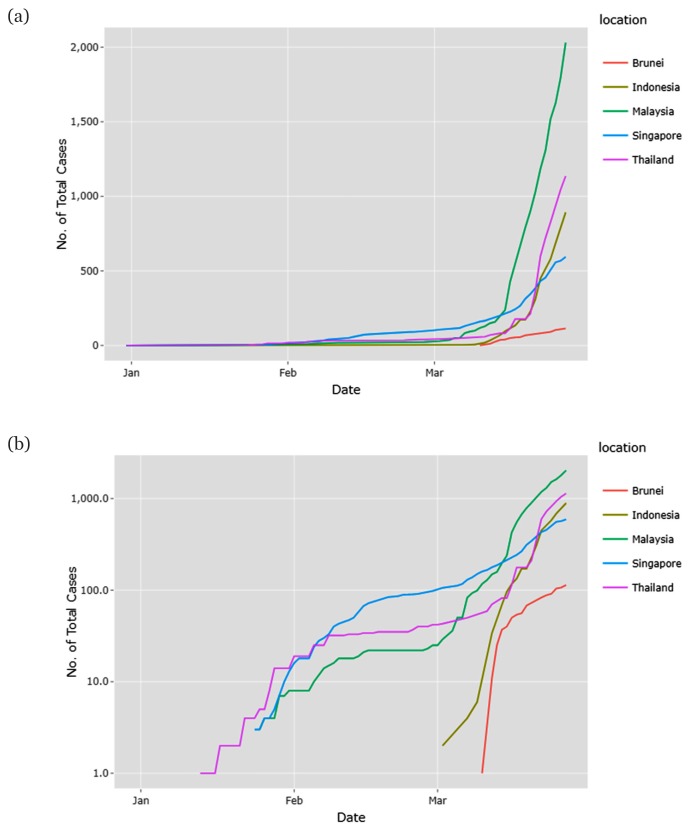
Trajectory of the cumulative number of COVID-19 cases in a (a) linear scale and a (b) log scale. The increase in cumulative COVID-19 cases slowed between January 2020 and late February 2020. However, in early March 2020, the case trajectory in these five countries increased significantly 1(a). The increases were most stark from mid-March to 28 March 2020. The trend on the log-scale 1(b) indicates an exponential growth in cumulative cases for three countries—Thailand, Malaysia and Singapore—which was steeper between mid-January and mid-February and then reduced until early March. Overall, the log-scale 1(b) shows two trajectories: a lower trajectory (Brunei and Singapore) and a higher trajectory (Thailand, Malaysia and Indonesia) (3)

**Figure 2:**
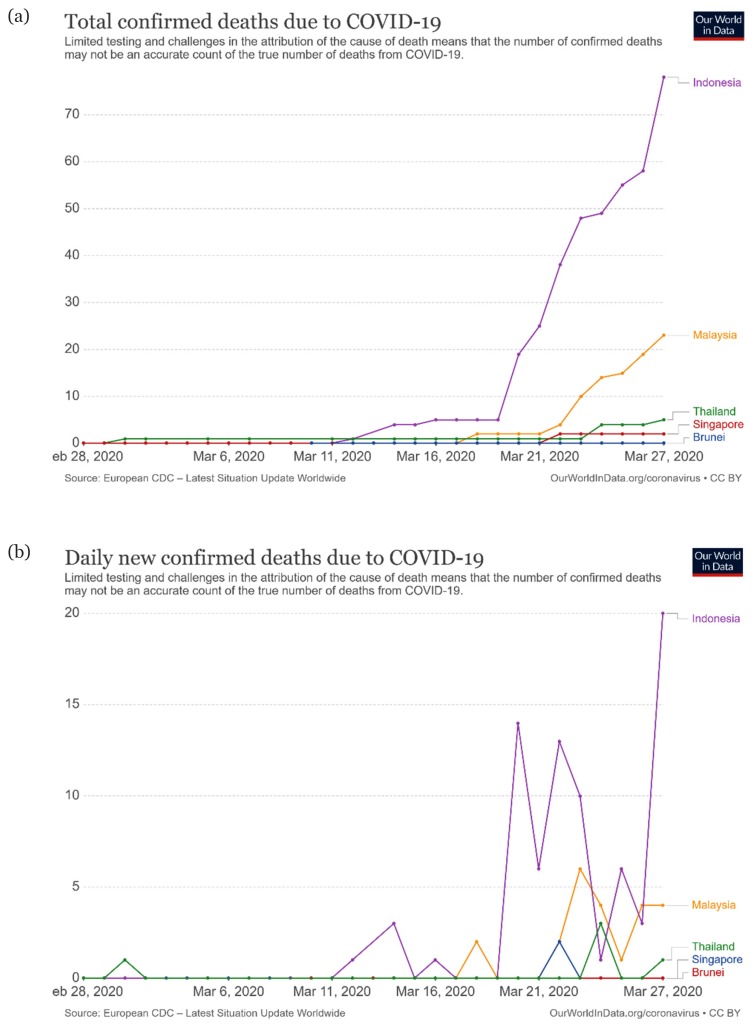
(a) Trajectory for cumulative COVID-19-related deaths and (b) daily reported number of COVID-19-related deaths. Thailand reported its first fatality in February 2020 and other countries (except for Brunei) began to report their first fatalities in mid-March 2020. Indonesia has had the largest number of fatalities (70 as of 27 March 2020), and the lowest were in Singapore (two deaths) and Brunei (no deaths). All countries reported fluctuating fatality numbers (except for Singapore) (1)

As of 26 March 2020, 171 countries have been affected by the coronavirus disease 2019 or known as COVID-19 pandemic with 467,866 infected patients and 20,845 deaths, representing a 4.5% mortality rate (1). While, at the time of writing, China is the most affected country, the rate of infection in China has slowed down significantly; however, this is not the case in Italy or the United States, which are the second and third most affected countries, respectively. Association of Southeast Asian Nations (ASEAN) countries, including Malaysia, have not been spared from COVID-19, although there have been fluctuations in the rate of infection demonstrated in the number of new cases and deaths reported in March 2020 (Table 1) (2). Barely three months after the first reported case, many countries have ordered lockdowns, including Malaysia. The Malaysian government announced an initial movement restriction order on 16 March 2020 and an extension to mid-April. These measure have been enacted for good reason. The trend in new cases does not appear to have peaked yet for Malaysia or other ASEAN countries (Figure 1) (3), although there have been fluctuations in the death rates (Figure 2) (1) perhaps due to different critical care capacities and resources in these countries.

COVID-19 is likely a zoonotic infection, which was transmitted from an unknown animal or environmental source to humans. It is now spreading via human-to-human transmission with an average basic reproductive rate (R_0_) of 2.2. In other words, for each patient, 2.2 other individuals become infected (4). For comparison, the R_0_ for severe acute respiratory syndrome (SARS) was estimated at 3.0 and Middle East respiratory syndrome (MERS) was less than 1.0. Based on the epidemiological data from Wuhan, there are several notable differences between COVID-19 and SARS and MERS: i) case fatality rates are lower; ii) asymptomatic spread can occur and iii) fever is more frequently absent (5). Gastrointestinal complaints are uncommon among COVID-19 patients but loss of smell and loss of taste have been increasingly reported as early signs of infection. Severe acute respiratory illness (SARI) is a severe complication, and typical computed tomography (CT) features include ground-glass and consolidative opacities in the periphery (6). However, it must be borne in mind that SARI can also be a manifestation of respiratory illnesses with other infectious or non-infectious causes, which need to be ruled out before attributing the presence of SARI to COVID-19.

## Halting the Spread with Rapid Diagnosis

**Figure 3:**
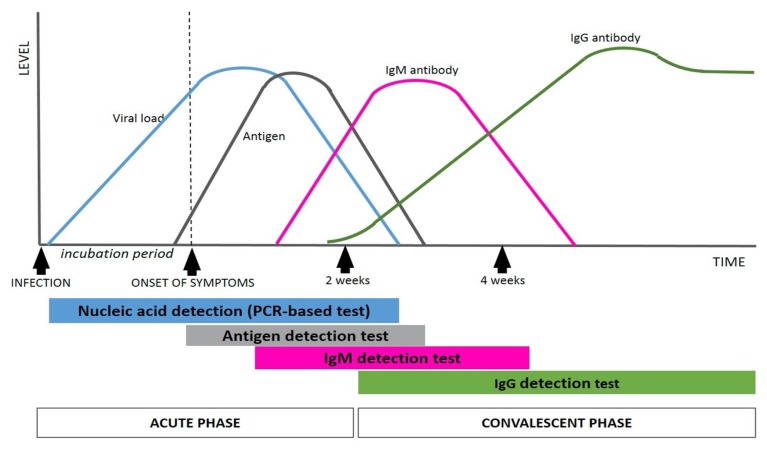
Diagnostic coverage of various available tests for COVID-19 from the acute phase to the convalescent phase

A critical factor in slowing down the pandemic is the rapid diagnosis of new cases. Nucleic acid amplification tests, such as real-time reverse transcription polymerase chain reaction (RT-PCR), provide the earliest and most accurate diagnosis, but they are costly and time consuming (7). Point-of-care tests, e.g. lateral flow assays for the detection of antibodies are more ideal in the field; however, these tests are of limited value due accuracy issues and the time required to obtain a diagnosis. Rapid antigen detection tests are still undergoing evaluation, and their efficacy is yet to be proven. Figure 3 illustrates various tests being evaluated for COVID-19 and their diagnostic coverage from the acute phase to the convalescent phase.

The sources and accessibility of specimens are also critical for diagnosis. For upper respiratory specimens, the viral Ribonucleic acid yield from nasopharyngeal swabs seems to offer more accurate results than oropharyngeal swabs. The SARS-CoV-2 virus is also detectable in blood, urine and stool; such specimens are not as reliable for diagnosis but are important from the transmission point of view. Proper transport and handling of specimens is necessary to ensure the integrity of the viral RNA and, hence, the accuracy of the diagnostic test. Adherence to biosafety practices is essential, and any testing should be performed in appropriately equipped laboratories by staff trained in the relevant technical and safety procedures (World Health Organization, 2020) (8). Non-propagative diagnostic laboratory work should be conducted at a biosafety level 2 (BSL-2) facility, whereas propagative work, including virus culture and isolation, should be conducted at a containment laboratory with inward directional airflow (BSL-3). Virus isolation is not routine but necessary for characterisation and to support the development of vaccines and other therapeutic agents.

## Challenges to Healthcare Workers

Until a vaccine is developed, healthcare workers who are in close contact with a suspect or a person under investigation (PUI) are at high risk of exposure to the virus. Other than doctors at the frontlines, i.e. screening and critical care units, certain specialties may have increased risks of aerosolised exposure, including respiratory medicine; otolaryngology; ophthalmology; and dental services. Many non-urgent procedures have been cancelled or postponed, including non-emergency surgeries. Other than the aerosolised risk, the virus may contaminate the operating theatre during surgery. Many learned societies, including the Academy of Medicine of Malaysia, have started releasing statements and guidelines to assist their members (9).

Meanwhile, grey areas and dilemmas exist without clear consensus, including guidelines for cancer patients on chemotherapy or those needing early surgical treatments. Furthermore, other issues should be addressed by hospitals through a management protocol or algorithm in place for frontline workers (10). For example, although many resources are being diverted to the frontlines and critical care, they will be inadequate during the height of the pandemic. Thus, there is an ethical but pragmatic challenge in the allocation of critical resources, such as intensive care unit (ICU) beds and ventilators (11). Age is often the sole criterion used for decision-making regarding ventilation, which can be morally difficult for people in general. Therefore, it is imperative that such decisions be made by a different team, and that the triage algorithm be reviewed regularly.

## Flattening the Curve

**Figure 4:**
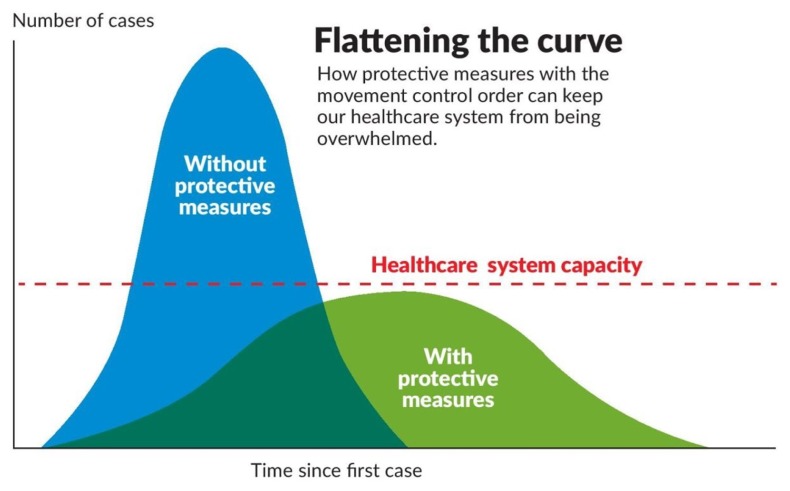
Movement restriction is critical to battle the pandemic within healthcare system capacity (15)

Although various antiviral agents such as avipiravir, ritonavir/lopinavir and hydroxychloroquine and other therapeutics, e.g. steroids and vaccines, are currently being considered to reduce the complications associated with COVID-19, including SARI and cytokine storms (12–14), the most cost-effective methods remain the public health approaches of contact tracing, isolation and social distancing. The COVID-19 pandemic is predicted to run a protracted course, and available healthcare resources are likely unsustainable; hence, we need to flatten the curve with lockdown measures (Figure 4) (15). However, the imposition of a lockdown needs to consider the sociocultural and economic factors of each country.

Extreme measures, such as the Wuhan closure, will not work in many countries, including Malaysia. Instead, many countries may look to the South Korean model as a more suitable alternative. In South Korea, rapid testing to rule out COVID-19 has been the key to its success, along with using personal information to track the spread of the virus (16). In Malaysia, where social gathering is a norm, the initial days of the movement restriction order have been extremely difficult but necessary so that containment measures could be put into place quickly. The current mitigation phase is critical in order to create a small window of opportunity to break the transmission of the virus to the larger community. To do this, everyone should be transparent about their history of travels, mass gatherings and any new symptoms. It is also important not to spread fake news to prevent panic and anxiety among the public.

The most common psychological reactions to a pandemic are fear and anxiety-related symptoms, such as panic, worry and emotionally distress (17, 18). While these reactions are expected, they can be overwhelming to those with pre-existing mental health issues (19) and can lead the public to panic buy excessive amounts of food or health-related items, such as masks and hand sanitizer (18). According the most recent data on COVID-19 study among 1,210 respondents from 194 cities in China (20), more than half of the respondents suffered moderate to severe psychological impacts; of those, 28.8% had moderate to severe anxiety, 16.5% had moderate to severe depressive symptoms and 8.1% had moderate to severe stress levels. The majority were worried about their family members and they spent at least 20 h per day at home (20).

The psychological sequelae of the pandemic is the emergence of fear and anxiety not only due to the disease itself but also due to the disruption of daily activities, social isolation caused by the restricted movement order and financial burdens, especially among those with low-income levels (21). For healthcare workers, the pandemic is exposing them to long-term stressors, which could impact their wellbeing (22) and lead to burnout due to increased job demands (such as increased workload and role conflicts) and reduced job resources which lead to loss of workplace control and autonomy (23). Early mitigation and psychological crisis interventions are already in place in Malaysia. Several government and non-governmental agencies are offering psychological first aid to the public through tele-counselling and hotline services. These interventions should be continued even after the pandemic is over, as data has shown that those who are affected by a pandemic still have varying degrees of stress disorders even after the event ends or they recover from the disease (24). In addition, clear information about the disease and progress updates on the situation could reduce the psychological impacts of COVID-19 among the public (20).

## Addressing the Socio-economic Impacts: Local Perspectives

**Figure 5:**
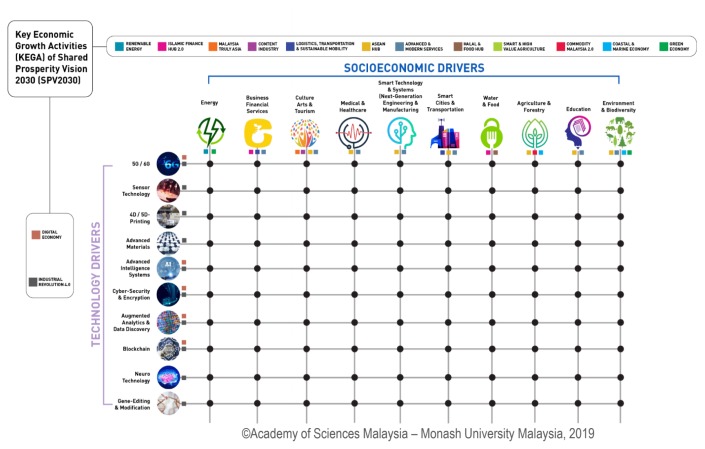
The Academy of Sciences Malaysia with assistance from Ministry of Health and the Monash University Malaysia, are exploring 10 technologies that will drive many aspects of Malaysia’s socio-economy, including preparation for future pandemics (25)

There is no doubt that the imposed movement control order in Malaysia will have adverse economic impacts; however, the government has been quick to respond by announcing an economic stimulus plan to weather this difficult period. Numerous important bodies, including the Academy of Sciences Malaysia, the Ministry of Health and the Ministry of Higher Education, are exploring the technology drivers of the present and the future to prevent and manage future epidemics and pandemics (Figure 5). Weaknesses have been identified and improvements are being explored before government action plans are made (25).

Another impact is on day-to-day work. Since many people are working from home, there is an urgent need to make better internet and software applications available for virtual meetings. From the healthcare perspective, telemedicine will play a greater role. Stable patients needing regular follow-ups, as well as patients in clinical trials, can be directed to tele-health services. These services could even be used for COVID-19 patients as a form of ‘forward triage’ before they arrive at emergency departments (26).

In addition, this unique crisis has provided an opportunity to improve online education from home. Almost 5 million school students and 1.2 million university students (including about 130,000 international students) in Malaysia have been affected by closures (27). Distance learning is not new for Malaysians and can be traced back to the 1990s but times have changed with rapid advancements in technology and new teaching innovations. Due to COVID-19, online learning is no longer merely an option; however, adopting virtual technologies and ensuring the readiness of students and institutions can be challenging (27). In this regard, Universiti Sains Malaysia (USM) is taking a leadership role as a model for other learning institutions in the country.

Finally, there has been a rise in research and collaborative opportunities during the COVID-19 pandemic (28). This is the time when scientists and clinicians should come out from their shining silos to work together and share their research data. Doing so will lead to more effective curbing of the disease and more rapid advancements in viral therapeutics. Therefore, we, the editorial members, urge the scientific community and the public to rise to the challenge, change their mindsets and stay safe and healthy whether you are at home or at work.

## Figures and Tables

**Table 1:** Number of new cases, new deaths, total cases and total deaths in South East Asia region (23–27 March 2020)

Date	Country	New cases	New deaths	Total cases	Total deaths
27/3/2020	Malaysia	235	4	2031	23
27/3/2020	Thailand	91	1	1136	5
27/3/2020	Indonesia	103	20	893	78
27/3/2020	Singapore	26	0	594	2
27/3/2020	Brunei	7	0	114	0
26/3/2020	Malaysia	172	4	1796	19
26/3/2020	Thailand	111	0	1045	4
26/3/2020	Indonesia	104	3	790	58
26/3/2020	Singapore	10	0	568	2
26/3/2020	Brunei	3	0	107	0
25/3/2020	Malaysia	106	1	1624	15
25/3/2020	Thailand	107	0	934	4
25/3/2020	Indonesia	107	6	686	55
25/3/2020	Singapore	49	0	558	2
25/3/2020	Brunei	13	0	104	0

Source: European Centre for Disease Prevention and Control (2)
